# Synthesis and Characterization of Short α and β-Mixed Peptides with Excellent Anti-Lipase Activities

**DOI:** 10.3390/molecules29040765

**Published:** 2024-02-07

**Authors:** Naeem Ahmed, Sabahat Asif, Muhammad Arfan, Qaiser Mahmood, Amjad Islam, Mansour K. Gatasheh, Muhammad Zia

**Affiliations:** 1 Department of Chemistry, School of Natural Sciences, National University of Sciences and Technology, Islamabad 44000, Pakistan; naeem.ahmed@sns.nust.edu.pk; 2 Department of Chemistry and Chemical Engineering, Syed Babar Ali School of Science and Engineering, Lahore University of Management Sciences (LUMS), Lahore 54792, Pakistan; sabahat6512@gmail.com; 3 Chemistry and Chemical Engineering Guangdong Laboratory, Shantou 515031, China; qaiser@ccelab.com.cn; 4 Key Laboratory for Preparation and Application of Ordered Structured Materials of Guangdong Province, College of Chemistry and Chemical Engineering, Shantou University, Shantou 515063, China; amjad@stu.edu.cn; 5 Department of Biochemistry, College of Science, King Saud University, P.O. Box 2455, Riyadh 11451, Saudi Arabia; mgatasheh@ksu.edu.sa; 6 Department of Biotechnology, Quaid-i-Azam University, Islamabad 45320, Pakistan; m.zia@qau.edu.pk

**Keywords:** β-amino acid, α and β-hybrid/mixed peptides, Arndt–Eistert method, Wolff rearrangement, lipase, obesity

## Abstract

Obesity is a source of significant pathologies and deadly diseases, including heart disease, diabetes, and cancer. One of the most intriguing strategies in the hunt for new anti-obesity medications is the inhibition of pancreatic lipase (PL). This study presents a novel application of short α and β-mixed peptides as pancreatic lipase inhibitors. These peptides were synthesized in the solution phase and characterized using FTIR and ^1^H-NMR. L-proline is present in a high percentage of natural anti-lipase peptides and was used as a β-amino acid in this study to enhance anti-lipase activity and proteolytic stability. Moreover, L-α-proline was converted to β-amino acid derivatives using the Arndt–Eistert method with the advantage of stereo control at the α-carbon. The synthesized peptides with anti-lipase activity are N**-**Boc-β-Pro-Gly-OBz (93%), N-Boc-O-Bz-Tyr-β-Pro-β-Pro-Gly-OBz (92%), N-Boc-O-Bz-Tyr-β-Pro-COOH (91%), N-Boc-Phe-β-Pro-OCH_3_ (90%), and N-Boc-O-Bz-Tyr-β-Pro-OCH_3_ (89%). These peptides may function as lead molecules for further modification to more significant molecules, which can help control obesity.

## 1. Introduction

Obesity is a complex disorder with excess fat deposits all over the body, especially in the abdomen. The World Health Organization (WHO) defines a person with a body mass index (BMI) equal to or greater than 25 as overweight and a BMI equal to or greater than 30 as obese. In 2021, 1.9 billion adults were heavy, and 650 million were obese. According to the global burden of disease, over 4 million people die each year due to this widespread disease [1]. Notably, obesity is a healthcare and socioeconomic disadvantage due to associated health issues. It may multiply the risk of sleep apnea, dyslipidemia, type-II diabetes, chronic renal disease, infertility, osteoarthritis, stroke, cardiovascular disease, non-alcoholic fatty liver disease, hypertension, and some forms of cancer [2,3]. Therefore, obesity control could reduce the mortality rate and occurrence of metabolic syndrome [4].

Weight management plans, specific diets like calorie restriction and intermittent fasting, weight reduction devices, weight loss medications, bariatric surgery, and liposuction are frequently available therapeutic strategies for overweight and obese people in modern society [5]. However, methods of losing weight, including liposuction or bariatric surgery, expose patients to high risks associated with surgery and, in some circumstances, even death [6].

Isolated compounds and plant extracts from natural sources have also been reported for their pancreatic lipase inhibitory potential, including chitosan [7], dietary fibers from cholestyramine and wheat bran [8], polylysine [9], protamine [10], and soya proteins [11]. Moreover, synthetic compounds are also reported as lipase inhibitors, e.g., Oxadiazol-2-ones [12], 5,7-dichloro-1,3-benzoxazole [13], derivatives of benzimidazole [14], chalcone [15], and quinolones [16]. Many natural and synthetic pancreatic lipase inhibitors are undergoing clinical trials. Still, these have yet to enter the clinical level because results in human trials compared to in vitro or in vivo studies were not significantly effective [17].

Due to serious side effects, most lipase inhibitor drugs have been withdrawn from the market [18]. These include phentermine [19], rimonabant [20]**,** and sibutramine [21]. Currently, orlistat [22] is the only FDA-approved drug available on the market to treat obesity. Long-term use of orlistat to treat obesity results in severe gastrointestinal side effects. These include liquid stool, diarrhea, oily spotting, flatulence, abdominal cramping, and fecal urgency [23]. Hence, novel pancreatic lipase inhibitors are urgently needed, with no severe side effects and long-term weight loss.

Recently, α-ketoamides, tripeptides, and 1,3-diketoamides have been shown as a few examples of amide functionality-based molecules with the potential for PL inhibition. Because of the higher reactivity of their carbonyl group structures with peptide functionality, they possess a more significant potential to inhibit PL. Similarly, lipase inhibitory peptides have been reported as from natural sources [24,25]. However, their progress is hindered due to increased proteolytic instability, poor bioavailability, and low yield [26].

Earlier research has proved the use of β-amino acids in peptides to design potent, selective, and stable molecules. Due to extra methylene in the backbone, α, β-mixed and β-peptides may adopt multiple conformations, may possess secondary structures, and are not identified by peptidases easily [27,28]. Hence, peptides with β-amino acids are expected to be more stable than α-peptides in vitro and in vivo.

In this study, we reported the design and synthesis of short αβ-mixed peptides. These peptides were synthesized through the solution phase and were evaluated for anti-lipase activity. The α-amino acid was converted to the β-amino acid derivative using the Arndt–Eistert method [29] and Wolff rearrangement [30] with the advantage of stereo control and high yield.

## 2. Results and Discussion

### 2.1. Chemistry

**Scheme 1:**
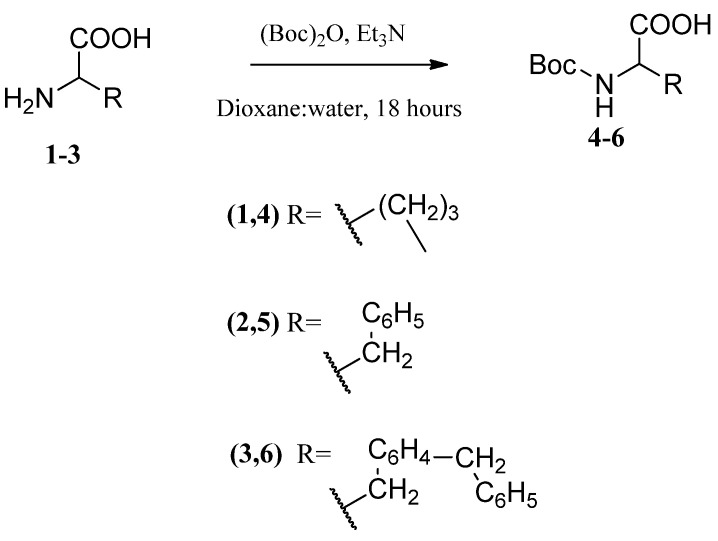
Synthesis of Boc-Protected Amino acids.

α-Amino acids and their derivatives, like L-proline **1**, L-phenylalanine **2**, and L-O-benzyl-protected tyrosine **3**, were treated with (Boc)_2_O in a water/dioxane (1:1) mixture to obtain N-Boc-L-Proline **4**, N-Boc-L-Phenylalanine **5**, and N-Boc-O-bz-Tyrosine **6**, respectively. The protection of compounds **4**–**6** was successful, indicated by characteristic bands in FTIR spectra ranging between 1666 and 1613 cm^−1^ (C=O carbamate) and between 1380 and 1361 cm^−1^ (C(CH_3_)_3_) from Boc (Scheme 1). Compound **6** was activated with ethyl chloroformate in the presence of triethylamine and converted to diazoketone **7** via the mixed anhydride method using diazomethane [29]. With the Wolff rearrangement [30], using triethylamine, silver benzoate, and dry methanol, compound **7** was converted to β-ester **8**. The Wolff rearrangement converts the diazo molecule to a carbonyl molecule by removing nitrogen and rearranging the carbene intermediate with the retention of the configuration [31].

**Scheme 2:**
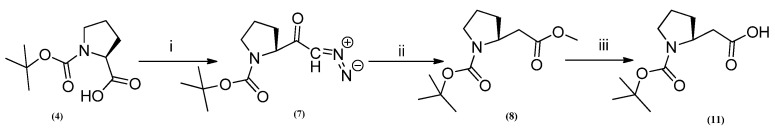
Reagents and conditions; (i) Ethyl chloroformate, Dry Et_3_N, Dry THF, under N_2_, −15 °C to −5 °C, CH_2_N_2_ in ether, 4 h (ii) Ag+, Dry Et_3_N, dry CH_3_OH, −25 °C to RT, under dark, 3 h (iii) 0.75N NaOH, Dry MeOH, 0^o^C to RT, 3–4 h.

In ^1^HNMR spectra, a singlet at δ 6.1 ppm (-CH=N_2_) integrated for one proton confirmed the formation of diazoketone **7**, and a singlet at δ 3.59 ppm (-OCH_3_) combined for three protons confirmed the formation of β-methyl ester of Boc proline **8**. Compounds **8**, **8A**, and **12** were Boc-deprotected with TFA in dry DCM [32]. Compound **8** was converted to β-acid **11** by base hydrolysis, as indicated by a shift in carbonyl IR band from 1736 cm^−1^ in **8** to 1727 cm^−1^ in **11** and confirmed by the disappearance of the singlet at δ 3.59 ppm (-OCH_3_) integrated for three protons and by the appearance of a broad singlet at δ 12.19 ppm (-COOH) in the ^1^HNMR spectra of **8** and **11**, respectively (Scheme 2).

**Scheme 3:**
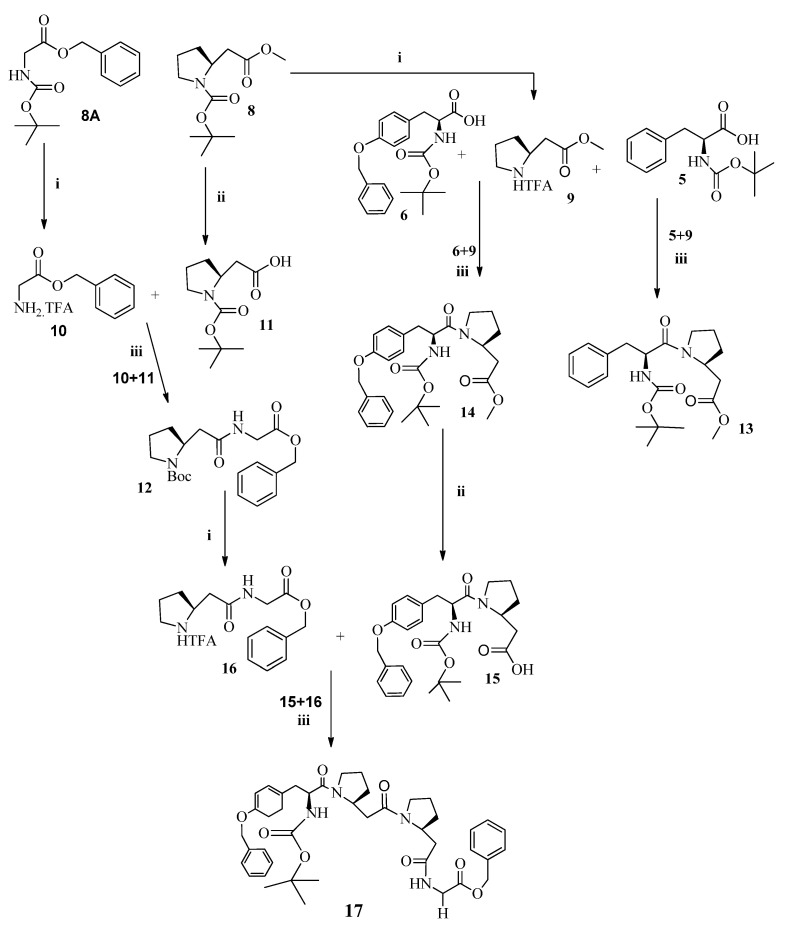
Reagents and conditions: (i) 0.75 N NaOH, Dry MeOH, RT, 8hr; (ii) TFA, Dry DCM, RT, 6hr; (iii) EDC, HOBt, Dry TEA, Dry CHCl_3_, 16hr

The synthesis of peptides **12**, **13**, **14**, and **17** was achieved using EDC as a coupling agent and HOBt in dry chloroform [33]. In the ^1^HNMR spectrum, a singlet at δ 1.39 ppm integrated for nine protons (C(CH_3_)_3_) from the Boc protecting group and another singlet at δ 7.37 ppm (-NH) integrated for one proton confirmed the synthesis of dipeptide N-Boc-β-Pro-Gly-OBz **12**. A singlet at 1.38 ppm integrated for nine protons (C(CH_3_)_3_) and one more singlet at 3.63 ppm combined for three protons (-OCH_3_) in the ^1^HNMR spectrum of dipeptide **13** confirmed its synthesis. In the ^1^HNMR spectrum, a singlet at 5.1 ppm integrated for two protons (-CH_2_-Ar monosubstituted) and another singlet at 3.6 ppm integrated for three protons (-OCH_3_) confirmed the synthesis of dipeptide **14**. The synthesis of tetrapeptide **17** was established by the presence of a singlet at 1.39 ppm integrated for nine protons (C(CH_3_)_3_) and a broad singlet at 6.95 ppm (-NH) combined for one proton in its ^1^HNMR spectrum (Scheme 3). It is evident that our desired α, β-mixed peptides were synthesized successfully with good yield.

### 2.2. Pancreatic Lipase (PL) Inhibitory Activity of Peptides

Lipases are essential in maintaining lipid homeostasis and in diseases like obesity that are linked to it. They hydrolyze dietary lipids into free fatty acids and simple glycerides, which help the body absorb them. The PL, also known as triacylglycerol esterase, is one of the most essential lipases for the intestinal absorption of fatty acids. In human PL, Ser-152, His-263, and Asp-176 make up a typical catalytic triad, which is highly conserved [34]. The problem of obesity can be controlled by inhibition of PL activity. In this study, the short α, β-mixed peptides (**12**, **13**, **14**, **15**, **17**) with 2–4 amino acid residues were synthesized through the solution phase, characterized by FTIR and ^1^HNMR spectral analysis, and for the first time screened for PL enzyme inhibition in vitro (Table 1). The results of the PL inhibition showed that all synthesized short α, β-mixed peptides were excellent inhibitors of PL, and their activity was also comparable to that of orlistat (standard drug). Peptide **12** presented the highest activity, followed by **17**, **15**, **13**, and **14** in decreasing order of inhibitory effect (92, 91, 90, and 89%, respectively). In the synthesized peptide set, **14** showed the weakest inhibitory potential (89%), 4% less than the peptide showing the highest inhibitory potential, **12**. This suggests that α, β-mixed peptides could be a good source of enzyme inhibitory peptides.

Published literature regarding α and β-mixed peptides as lipase inhibitors is not available. This is the first study to report α and β-mixed peptides as PL inhibitors. However, peptides incorporating β-amino acids have been reported as anti-HIV [35], anti-cancer [36], antimicrobial [37], and anti-bacterial [38]. Recently, plant sources [39,40] have been extensively studied as PL inhibitors. In addition, synthetic PL-inhibiting molecules like thiazole–benzimidazole conjugates [41], quinazolinone hybrid analogs [42], coumarin derivatives [43], indole–thiazolidinedione hybrids [44], and salicylanilides [45] have also been reported. However, numerous studies, such as (Mudgil, 2022) [25] (Ketprayoon, 2021) [46] and (Esfandi, 2021) [47], have investigated the anti-lipase activity of peptides from protein sources.

The synthetic peptides derived from β-amino acids in this study exhibited a higher activity (89 to 92%) than that found in earlier studies of α-peptides. For example, the peptides ELPPHFL, APFPLR, and LNFEPR from sea cucumber (*C. frondose*) had lipase inhibition percentages of 75, 60, and 72%, respectively [48]. The three long chemically synthesized α-peptides with 17–23 residues FFRSKLLSRGAAAAKGALLPQYW, RCMAFLLSDGAAAAQQLLPQYW, and RPAQPNYPWTAVLVFRH from cumin seeds showed inhibition percentages of 55, 50, and 23%, respectively. Moreover, isolated α-peptides LAPSTIK and IIAPPER [49] from *Gryllodes sigillatus*, HLPGRG [25] from cow casein hydrolysates, and PAGNFLP [25] from camel casein hydrolysates also exhibited PL inhibition. Zielinska et al. reported the anti-lipase activity of peptides from the in vitro digestion of edible insects [49]. The author reported two α-peptides, ILAPPER and FDPFPK, with potent lipase inhibitory activities. Moreover, both peptides of this study have two proline residues at different positions. The author further concluded that Pro and Leu might contribute to the activity. Similarly, Zhang (2022) [48] reported that the α-peptide APFPLR, derived from sea cucumber (*C. frondose*), with Pro, Leu, and Phe residues, exhibited anti-lipase activity. The sequence of peptides synthesized in this study also has Tyr, Phe, Leu, Gly, and Pro.

The short peptides of our study showed excellent PL inhibition potency compared to α-peptides in the literature [48,50] and comparable activity to that of a standard drug (orlistat), which is encouraging. On the other hand, the long-term use of orlistat results in severe side effects [51,52]. Furthermore, the presence of the β-amino acid in our synthesized short α, β-mixed peptides can be a better choice as an anti-lipase inhibitor because the peptidic nature will result in fewer side effects. Therefore, short α and β-mixed peptides can be lead compounds for developing anti-lipase drugs more effective than drugs already present in the market with many side effects. 

The significance of this work is the synthesis of short peptides with β-amino acids and their novel application as PL inhibitors. The peptides from natural sources used for comparison in this study have six to twenty-three amino acids. But peptides of this synthesis are short, with two to four amino acids, are more active, and are more economical to synthesize. Moreover, the peptides from natural sources are α- in nature with a short half-life both in vitro and in vivo [53,54].

## 3. Materials and Methods

### 3.1. General

All reagents were purchased from Sigma Aldrich (St. Louis, MO, USA)/Merck (Lowe, NJ, USA). The melting point (m.p.) was determined on a Staffordshire ST15 OSA, UK, capillary melting point apparatus. The optical rotation of the synthesized compounds was measured by using an ATAGO (Tokyo, Japan) automatic polarimeter at 589 nm and 20 °C temperature (concentration in 0.01 g/1 mL CH_3_OH). FTIR analysis of the synthesized compounds was performed using Bruker Alpha Platinum-ATR (Berlin, Germany). ^1^H NMR spectra were recorded using a Bruker AC 300 NMR spectrometer. CHNS analysis of all compounds was performed using a CKTC-SECHN200 CHNS analyzer. During synthesis, dry solvents were operated using the following methods:

Chloroform: To dry chloroform, 5 g of P_2_O_5_ was added to 100 mL of the solvent in a round-bottom flask. The mixture was refluxed for 4–5 h, and after cooling, the solvent was distilled under nitrogen. 

Dichloromethane: DCM was distilled using the same method as described for chloroform. 

Triethylamine**:** The required amount of triethylamine was dried by adding KOH, refluxing for 4 h, cooling to room temperature, distilling under an inert atmosphere, and storing it over KOH pellets. 

Tetrahydrofuran: To dry tetrahydrofuran (THF), CaH_2_ was added and drying tubewas attached to a reflux condenser. It was stirred overnight to remove as much H_2_O as possible. Then, in two neck flasks, THF was collected by filtration, and benzophenone and sodium metal were added after a few intervals until the blue color persisted for a more extended period. Finally, it was distilled under nitrogen and stored over sodium wire. 

Diethyl ether: Diethyl ether was dried using the same method described above for tetrahydrofuran. 

Methanol: Methanol was dried by adding 5 g of Mg and turning it to 100 mL. A few crystals of iodine were added and refluxed until white turbidity appeared. After cooling, it was distilled under nitrogen and stored in a brown bottle [55].

### 3.2. NH_2_-Group Protection of L-Amino Acids *(**4**–**6**)*

L-amino acids (**1**–**3**) (13.3 mmol) were dissolved in a 1:1 dioxane/water (32 mL) mixture. Then, triethylamine (1.6 eq, 21.3 mmol, 2.97 mL) was added, followed by di-tert-butyl dicarbonate (1.1 eq, 14.6 mmol, 3.19 g) at 0 °C, and the reaction mixture was stirred at room temperature for 18 h. After reaction completion, checked by TLC, the reaction mixture was concentrated. To this, ethyl acetate and water were added, and the acidic condition (pH = 1) was maintained using 1 M KHSO_4_. After ethyl acetate extraction, the organic layer was washed with brine, dried over MgSO_4_, and rotary evaporated to yield N-Boc-L-amino acids [56] (**4**–**6**).

- N-(Boc)-L-Proline (**4**)

Reaction time: 18 h; physical appearance: light-yellow crystals, Melting point: 136 °C; yield: 98%; TLC (system: 20% EtAc/n-hexane, stain: Ninhydrin); [α]_D_^25^: +7.1° (c = 1 mg/10 mL CH_3_OH); FTIR (neat, v_max_): 2873(C-H, CH_2_, str), 2968(C-H, CH_3_, str), 1734(C=O, acid), 1632(C=O, carbamate), 1422(C-N, carbamate), 1361(C(CH_3_)_3_), 1159(C-O) cm^−1^. Anal. Calcd for C_10_H_17_NO_4_ (215.25): C, 55.80; H, 7.96; N, 6.5. Found: C, 55.78; H, 7.98; N, 6.51.

- N-(Boc)-L-Phenylalanine (**5**)

Reaction time: 18 h; physical appearance: thick liquid; TLC (system: 30% EtAc/n-hexane, stain: Ninhydrin); yield: 92%; [α]_D_^25^: +25° (c = 1 mg/mL CH_3_OH); FTIR (neat, v_max_): 3330(sec. N-H, str), 3050(Ar-C=CH), 2979(C-H, CH_3_, str), 2930(C-H, CH_2_, str), 1714(C=O acid), 1454(Ar-C=C), 1403(C-N, carbamate), 1368(C(CH_3_)_3_), 1279(C-O, acid), 1155(C-O, carbamate) cm^−1^. Anal. Calcd for C_14_H_19_NO_4_ (265.13): C, 63.38; H, 7.22; N, 5.28. Found: C, 63.37; H, 7.25; N, 5.27.

- N-Boc-O-Bn-L-Tyr (**6**)

Reaction time: 18 h; physical appearance: thick brown gel; yield: 97%; TLC (system: 30% EtAc/ n-hexane, stain: Ninhydrin); [α]_D_^25^: +27° (c = 2 mg/mL CH_3_OH); FTIR (neat, v_max_): 3262(sec. N-H, str), 3400–3200(O-H, acid), 3050(Ar-C=CH), 2978(C-H, CH_2_, str), 2930(C-H, CH_3_, str), 1613(C=O, carbamate), 1453(Ar-C=C), 1380(C(CH_3_)_3_), 1248(C-O, acid), 1155(C-O, carbamate)cm^−1^. Anal. Calcd for C_21_H_25_NO_5_ (371.43): C, 67.91; H, 6.78; N, 3.77. Found: C, 67.87; H, 6.76; N, 3.76.

### 3.3. Generation of Diazomethane Ethereal Solution 

The mini diazald apparatus from Aldrich was used to generate diazomethane from diazald. This apparatus is a single compact condenser, a reaction vessel with a receiver, and an additional funnel (with clear-seal joints). The cold finger is a unique feature of this apparatus; when filled with isopropyl alcohol/liquid nitrogen slush, it prevents the escape of diazomethane and ether into the environment efficiently.

The condenser was filled with isopropyl alcohol and liquid nitrogen to make slush to liquefy the generated diazomethane ether solution. Then, the condenser was attached to the receiving flask (with clear-seal joints) and cooled in an isopropyl alcohol/liquid nitrogen bath. A reaction vessel containing potassium hydroxide solution was supplemented with ether (10 mL) and 2-(2-ethoxyethoxy) ethanol (16 mL). Over the reaction vessel, a separatory funnel was attached (with clear-seal joints) and charged with a solution of diazald (5 g, 23 mmol) in ether (40 mL). The reaction vessel was warmed to 65 °C in a water bath, and diazald solution was added for over 40 min. The rates of addition and distillation were approximately kept the same. In addition, 20 mL of diethyl ether was added after the diazald was completely used, and the distillation continued until the distillate was colorless [57].

### 3.4. Synthesis of N-Boc-Proline Diazoketone *(**7**)*

Boc-L-Proline (**7**) (6.9 mmol, 1.5 g) was dissolved in dry distilled THF (34.9 mL), followed by the addition of Et_3_N (1.2 eq, 8.4 mmol, 1.2 mL). Ethyl chloroformate (1.5 eq, 10.5 mmol, 1 mL) was added, stirred at −15 °C under N_2_ for 20 min, and then treated with CH_2_N_2_ solution in ether. The solvent was evaporated, and the reaction mixture was dissolved in ethyl acetate. It was further washed with concentrated NaHCO_3_, NH_4_Cl, and NaCl solutions. The organic layer was evaporated under a vacuum to obtain impure Boc-Pro diazoketone as a dark-yellow gel. It was further purified by column chromatography using an eluent (7% ethyl acetate and n-hexane) to obtain pure Boc-Pro diazoketone (**7**) as a yellow gel.

Reaction time: 5 h; physical appearance: yellow gel; yield: 74%; TLC (system: 30% EtAc/n-hexane, stain: Ninhydrin); [α]_D_^25^: −11.2° (c = 2 mg/mL CH_3_OH); FTIR (neat, v_max_): 2974(C-H, CH_3_, str), 2971(C-H, CH_2_, str), 2105(-C=N_2_), 1680(C=O, carbamate), 1626(N-H, bending) 1360(C(CH_3_)_3_), 1155(C-O) cm^−1^. ^1^H NMR (300MHz, DMSO): δ 6.1 (1H, bs), 4.17 (1H, dd, *J* = 9.6, 7.5 Hz), 3.30–3.29 (2H, m), 2.15–2.10 (1H, m), 1.81–1.78 (3H, m), 1.33 (9H, s) ppm. Anal. Calcd for C_9_H_14_N_3_O_3_^+^ (212.23): C, 50.94; H, 6.65; N, 19.80. Found: C, 50.9; H, 6.68; N, 19.85.

### 3.5. Synthesis of N-Boc-Pro-β-Methyl Ester *(**8**)*

Boc-Pro diazoketone (**11**) (2.1 mmol, 0.5 g) was dissolved in dry CH_3_OH (6.16 mL) and then under N_2_ at −25 °C in dark silver benzoate (0.15 eq, 0.23 mmol, 0.053 g) dissolved in triethylamine (2.9 eq, 6.1 mmol, 0.8 mL) was added. After 3 h, the rotary reaction mixture evaporated, and the residue was dissolved in ethyl acetate. The organic layer was washed with NaHCO_3_, NH_4_Cl, and NaCl solutions, dried over MgSO_4,_ and evaporated. The crude product was purified by column chromatography using a 15% n-hexane/ethyl acetate solvent system [30].

Reaction time: 8 h; appearance: thick white gel; yield: 95%; TLC (system: 20% EtAc/n-hexane, stain: Ninhydrin); [α]_D_^25^: −11.2° (c = 2 mg/mL CH_3_OH); FTIR (neat, v_max_): 2973(sec. N-H, str), 2956(C-H, CH_3_, str), 2873(C-H, CH_2_, str), 1736(C=O, ester), 1688(C=O, carbamate), 1460(Ar-C=C), 1365(C(CH_3_)_3_), 1161(C-O) cm^−1^. ^1^H NMR (300 MHz, DMSO): δ 4.00–3.97 (1H, m), 3.59 (3H, s), 3.33–3.19 (2H, m), 2.72–2.65 (1H, m), 2.37 (2H d, *J* = 9.3 Hz), 1.80–1.60 (3H, m), 1.39 (9H, s) ppm. Anal. Calcd for C_12_H_21_NO_4_ (243.30): C, 59.24; H, 8.70; N, 5.76. Found: C, 59.21; H, 8.72; N, 5.74.

### 3.6. Boc-Deprotection of N-Boc-Amino Ester *(**9**)* and *(**10**)*

At 0 °C, N-Boc-Proline-β-methyl ester (1.23 mmol, 300 mg) was dissolved in dry dichloromethane (7 mL) under an inert atmosphere. After the addition of trifluoroacetic acid (TFA) (5.5 eq, 6.34 mmol, 0.52 mL), the reaction mixture was stirred for 3 h. After reaction completion, the solvent was evaporated to obtain the product [32].

### 3.7. Boc-Deprotection of N-Boc-Peptide Ester *(**16**)*

Under an inert atmosphere, N-Boc-β-Pro-α-Gly-OBn (500 mg, 1.049 mmol) was dissolved in dry DCM (6 mL) at 0 °C. Then, trifluoroacetic acid (TFA) (8.5 eq, 5.770 mmol, 0.44 mL) was added to the reaction mixture for 5 h. TLC indicated reaction completion, and rotary evaporation gave the final product [32].

### 3.8. Synthesis of N-Boc-β-Amino Acid *(**11**)*

At 0 °C, fully protected N-Boc-Proline-β-Methyl Ester was dissolved in MeOH and treated with 0.75 N NaOH (2 eq) solution portion-wise for 30 min. The reaction was completed in 8 h, as checked by TLC. The mixture was adjusted to pH 2–3 with 1 N HCl, extracted with ethyl acetate, dried over MgSO_4,_ and evaporated until dryness [29].

- (N-Boc-L-Proline-β-Acid) (**11**)

Reaction time: 10 h; physical appearance: white crystalline solid; melting point: 98° C; yield: 95%; TLC (system: 30% EtAc,/n-hexane, stain: Ninhydrin); [α]_D_^25^: −26° (c = 2 mg/mL CH_3_OH); FTIR (neat, v_max_): 2973(sec. N-H, str), 2919(C-H, CH_3_, str), 2873(C-H, CH_2_, str), 1727(C=O, acid), 1650(C=O, carbamate), 1361(C(CH_3_)_3_), 1159(C-O) cm^−1^. ^1^H NMR (300 MHz, DMSO): δ 12.19 (1H, bs), 3.91 (1H, dt, *J* = 8.7 Hz), 3.33 (2H, dd, *J* = 11.4, 3.5 Hz), 2.65–2.55 (1H, m), 2.26–2.16 (1H, m), 2.14–1.99 (1H, m), 1.97–1.94 (2H, m), 1.39 (9H, s) ppm. Anal. Calcd for C_11_H_19_NO_4_ (229.28): C, 57.63; H, 8.35; N, 6.11 Found: C, 57.61; H, 8.32; N, 6.09.

### 3.9. Synthesis of N-Boc-Peptide Acid *(**15**)*

To dry methanol, a fully protected N-Boc-peptide-β-methyl ester was added at 0 °C. In this mixture, 0.75 N NaOH (4.0 eq) solution was added in 30 min. The TLC indicated the completion of the reaction in 8 h, and then the reaction mixture was adjusted to pH 2–3 with 1 N HCl, and the product was extracted in ethyl acetate, dried over MgSO_4_, and finally evaporated to dryness [29].

### 3.10. General Procedure for the Synthesis of Short α,β-Mixed Peptides *(**12**–**14**)*

N-Boc β-amino acid was dissolved in dry CHCl_3_ (8 mL) and was treated with HOBt (1.2 eq, 1.25 mmol, 0.17 g) and EDC (1.2 eq, 1.25 mmol, 0.19 g). Boc-deprotected amino acid trifluoro acetate (1.04 mmol) was separately dissolved in dry chloroform (7 mL) at 0 °C under N_2_. Then, Et_3_N (6 eq, 11.6 mmol, 1.6 mL) was periodically added to it with constant stirring at 0 °C. Two solutions were then mixed, the reaction mixture was stirred for 16 h at room temperature, and reaction progress was monitored through TLC. After complete conversion, chloroform was added, and the organic layer was washed with 1 N HCl, NaHCO_3_, and NaCl and then dried over MgSO_4_ and rotary evaporated to give the respective peptides.

- Dipeptide (N-Boc-β-Pro-α-Gly-OBn) (**12**)

Reaction time: 16 h; physical appearance: light-yellow thick gel; yield: 75%; TLC (system: 3% CH_3_OH/CHCl_3_, stain: Ninhydrin); [α]_D_^25^: −26° (c = 2 mg/mL CH_3_OH); FTIR (neat, v_max_): 3306(sec. N-H, str), 3050(Ar-C=CH), 2966(C-H, CH_3_, str), 2875(C-H, CH_2_, str), 1748(C=O ester), 1668(C=O, amide), 1541(N-H, bend, amide), 1455(Ar-C=C), 1394(CH_3_, bend), 1365(C(CH_3_)_3_), 1255(C-O, ester), 1188(C-O, carbamate), 973(C-H, bend, mono sub), 915(C-H, bend, mono sub)cm^−1^. ^1^H NMR (300 MHz, DMSO): δ 8.37–8.35 (4H, m), 7.37 (1H, s), 5.125 (2H, s), 3.96 (2H, s), 3.96 (3H, d, *J* = 6.3 Hz), 3.4 (1H, bs), 3.33 (2H, d, *J* = 0.9 Hz), 3.30 (2H, d, *J* = 6.9 Hz), 1.9–1.6 (4H, m), 1.39 (9H, s) ppm. Anal. Calcd for C_20_H_28_N_5_O_5_ (376.45): C, 63.81; H, 7.50; N, 7.44. Found: C, 63.80; H, 7.51; N, 7.43.

- Dipeptide (N-Boc-α-Phe-β-Pro-OCH_3_) (**13**)

Reaction time: 12 h; physical appearance: white thick gel; yield: 70%; TLC (system: 3% CH_3_OH/CHCl_3_, stain: Ninhydrin); [α]_D_^25^: −6° (c = 2 mg/mL CH_3_OH); FTIR (neat, v_max_): 3300(sec. N-H, str), 2974(C-H, CH_3_, str), 2875(C-H, CH_2_, str), 1705(C=O ester), 1634(C=O, amide), 1500(N-H, bend, amide), 1434(Ar-C=C),1365(C(CH_3_)_3_), 1247(C-O, ester), 1162(C-O, carbamate)cm^−1^. ^1^H NMR(300 MHz, CDCl_3_): δ 7.3–7.25 (5H, m), 6.2 (1H, bs), 4.6 (1H, dd, *J* = 7.5 Hz), 4.34 (1H, t, *J* = 9 Hz), 3.63 (3H,s), 3.18–3.10 (1H, m), 3.05–2.94 (1H, m), 2.91 (2H, d, *J* = 3.6 Hz), 2.86 (2H, d, *J* = 3 Hz), 2.17–2.07 (3H, m), 1.82–1.80 (1H, d, *J* = 3.9 Hz), 1.38 (9H, s) ppm. Anal. Calcd for C_21_H_30_N_2_O_5_ (390.48): C, 64.60; H, 7.74; N, 7.17. Found: C, 64.62; H, 7.75; N, 7.18.

- Dipeptide (N-Boc-O-Bn-α-Tyr-β-Pro-OCH_3_) (**14**)

Reaction time: 16 h; physical appearance: brown thick gel; yield: 72%; TLC (system: 3% CH_3_OH/CHCl_3_, stain: Ninhydrin); [α]_D_^25^: +0.92° (c = 2 mg/mL CH_3_OH); FTIR (neat, v_max_): 3020(Ar-C=CH), 2927(C-H, CH_3_, str), 2875(C-H, CH_2_, str), 1733(C=O ester), 1635(C=O, amide), 1510(N-H, bend, amide), 1435(Ar-C=C), 1366(C(CH_3_)_3_), 1240(C-O, ester), 1165(C-O, carbamate), 800(para substitution)cm^−1^. ^1^HNMR (300 MHz,CDCl_3_): δ 7.79–7.76 (2H, m), 7.69–7.66 (2H, m), 7.5–7.3 (5H, m), 6.96 (1H, bs), 5.13 (2H, s), 4.48–4.44 (1H, t, *J* = 7.2 Hz), 4.27–4.20 (1H, m), 3.65 (3H, s), 3.66–3.65 (1H, m), 3.63–3.60 (1H, m), 2.89 (2H, d, *J* = 3.6 Hz), 2.87 (2H, d, *J* = 3 Hz), 2.1–2.05 (4H, m), 1.38 (9H, s) ppm. Anal. Calcd for C_28_H_36_N_2_O_6_ (496.60): C, 67.72; H, 7.31; N, 5.64. Found: C, 67.73; H, 7.32; N, 5.63.

### 3.11. Tetrapeptide (N-Boc-O-Bn-α-Tyr-β-Pro-β-Pro-α-Gly-OBn) *(**17**)*

Reaction time: 16 h; physical appearance: brown thick gel; yield: 70%; TLC (system: 10% CH_3_OH/CHCl_3_, stain: Ninhydrin); [α]_D_^25^: +40.59° (c = 2 mg/mL CH_3_OH); FTIR (neat, v_max_): 3000(Ar-C=CH), 2921(C-H, CH_2_, str), 2852(C-H, CH_3_, str), 1733(C=O ester), 1615(C=O, amide), 1510(N-H, bend, amide), 1455(Ar-C=C), 1377(C(CH_3_)_3_), 1240(C-O, ester), 1168(C-O, carbamate), 742(CH_2_, mono-substitution)cm^−1^. ^1^HNMR (300 MHz, CDCl_3_): δ 7.92 (2H, d, *J* = 8.4 Hz), 7.72 (2H, d, *J* = 8.5 Hz), 7.53–7.32 (6H, m), 7.22–7.19 (2H, m), 7.20 (1H, bs), 6.95 (1H, bs), 5.16 (2H, s), 5.13 (2H, s), 4.52–4.47 (1H, t, *J* = 7.5 Hz), 4.3–4.2 (2H, m), 4.09 (2H, s), 3.27–2.99 (10H, m), 2.1–2.05 (8H, m), 1.39 (9H, s) ppm. Anal. Calcd for C_28_H_36_N_2_O_6_ (496.60): C, 67.72; H, 7.31; N, 5.64. Found: C, 67.73; H, 7.32; N, 5.63.

## 4. Lipase Inhibition Assay

Lipase inhibition was performed to assess the anti-lipase activity. The lipase was taken in ultra-pure water (10 mg/mL) and centrifuged for 5 min at 16,000 rpm, and the fresh enzyme was made from its supernatant. The substrate utilized was olive oil. Sequentially, buffer (350 µL), lipase (150 µL), and test samples (50 µL) were added to Eppendorf tubes. To start the reaction, olive oil (450 µL) was introduced, and orlistat was used as a blocking agent. An Eppendorf tube with no sample, having a buffer, lipase, and substrate, functioned as a blank. The samples were incubated for 2 h at 37 °C and centrifuged for 1 min at 16,000 rpm; then, mixtures were taken in a 96-well plate, and at 400 nm, absorbance was measured as compared to standard (orlistat). The following formula was used to calculate the percentage of enzyme inhibition [58]:

% Enzyme Inhibition = OD (b) − OD (s) ÷ OD (b) × 100

where OD (b) = Absorbance value of blank; OD (s) = Absorbance of the test sample.

## Figures and Tables

**Table 1:** The anti-lipase activity of short α, β-mixed peptides**.**

Codes	Short α, β-Mixed Peptides	% Lipase Inhibition
**12**	N-Boc-β-Pro-Gly-OBz	93
**13**	N-Boc-Phe-β-Pro-OCH_3_	90
**14**	N-Boc-O-Bz-Tyr-β-Pro-OCH_3_	89
**15**	N-Boc-O-Bz-Tyr-β-Pro-COOH	91
**17**	N-Boc-O-Bz-Tyr-β-Pro-β-Pro-Gly-OBz	92
Standard	Orlistat	98

## Data Availability

Data are contained within the article.
